# The Recent Research Progress of the Tumor mRNA Vaccine

**DOI:** 10.3390/vaccines12101167

**Published:** 2024-10-12

**Authors:** Hao Zhao, Miying Li, Jiaren Zhou, Lidan Hu, Shaohong Lu, Pan Li

**Affiliations:** 1 Engineering Research Center of Novel Vaccine of Zhejiang Province, Hangzhou Medical College, Hangzhou 310051, China; zhaohao@hmc.edu.cn (H.Z.); 1109220805@hmc.edu.cn (M.L.); zhoujiaren@hmc.edu.cn (J.Z.); lsh@zjams.com.cn (S.L.); 2 Institute of Translational Medicine, Zhejiang University, Hangzhou 310058, China; hulidan@zju.edu.cn

**Keywords:** mRNA vaccine, tumor, immunotherapy, molecular structure

## Abstract

Tumors have long posed a significant threat to human life and health, and the messenger ribonucleic acid (mRNA) vaccine is seen as an attractive approach for cancer immunotherapy due to its developmental simplicity, rapid manufacture, and increased immune safety and efficiency. In this review, we have summarized details of the developmental history of mRNA vaccines, discussed the basic molecular structure and the effect on the stable and translation level of mRNA, analyzed the underlying immune efficiency and mechanisms on tumors, and assessed the current status of clinical research. We explored the treatment and application prospects of mRNA vaccines, aiming to provide perspectives on the future of mRNA tumor vaccines for ongoing clinical research.

## 1. Introduction

Cancer is a major social, public health, and economic problem in the 21st century, representing a significant threat to human life and health, with nearly 20 million new cases and 9.7 million cancer-related deaths occurring in 2022. It is estimated that approximately one-fifth of both men and women will be diagnosed with cancer in their lifetime, and about one-ninth of men and one-twelfth of women will succumb. Moreover, the annual incidence rate of new cancer cases is expected to reach 35 million by 2050 [1], underscoring the urgent need for novel tumor treatments that enhance safety and efficacy.

mRNA tumor vaccines as a promising platform for cancer immunotherapy are garnering widespread attention, and have the merits of high potency, safe administration and rapid development. mRNA tumor vaccines stimulate the body’s innate and adaptive immune response within delivered mRNA molecules, encoding antigens including cytokines and tumor-associated or tumor-specific antigens (TAAs or TSAs) to cells [2]. Within cells, the antigens present on the surface of antigen-presenting cells (APCs) of major histocompatibility complexes (MHCs) and then elicit robust T- and B-cell immune responses.

Up to now, mRNA vaccines have not only received approval in a remarkably short timeframe, but have also demonstrated excellent safety and efficacy in clinical trials [3]. Thus, mRNA vaccines are increasingly recognized within the realm of tumor immunotherapy and have progressed into the clinical development stage [4]. This review aims to comprehensively summarize the defining characteristics of mRNA vaccines and their research advancements in the rapidly evolving field of tumor immunotherapy.

## 2. Molecular Structure and Immunological Characteristics of mRNA Vaccines

**Figure 1:**
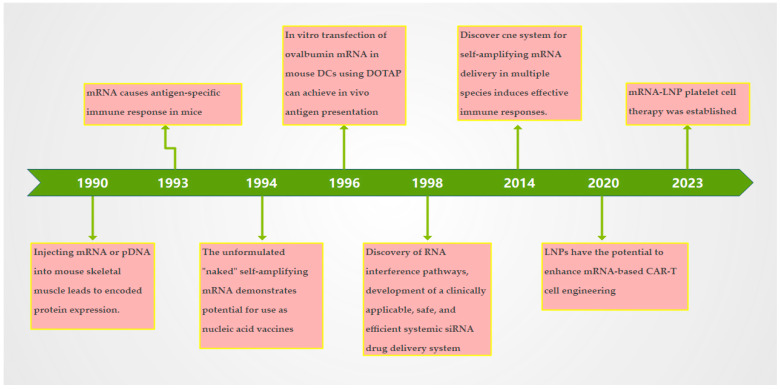
Time axis of mRNA vaccine development: summary of the time axis of mRNA vaccine development from 1990 to 2023. pDNA: Plasmid DNA; CNE: Cationic Nanoparticle Emulsion; LNP: Lipid Nanoparticle; DOTAP: N-1-(2,3-diethoxy) propyl-n, n, n-trimethylammonium sulfate; DC: Dendritic cells.

The studies on the aspects of mRNA stability, immunogenicity, targeting, and delivery systems to increase the level of immune response of mRNA vaccines were begun in 1990 [5,6] (Figure 1).

The pros and cons of mRNA vaccines include the fact that the exogenous mRNA can be directly translated into the desired protein through molecular modification, and various mRNA vaccines can be developed by altering the length or composition of the sequence, which effectively reduces both delivery time and production costs. Moreover, assisting the protective carriers such as lipid nanoparticles (LNPs) and viruses, means the life of naked mRNA is extended to a maximum of three months. Therefore, further research is needed to extend the shelf life of mRNA vaccines.

### 2.1. Molecular Structure and Its Modification of mRNA Vaccine

**Figure 2:**
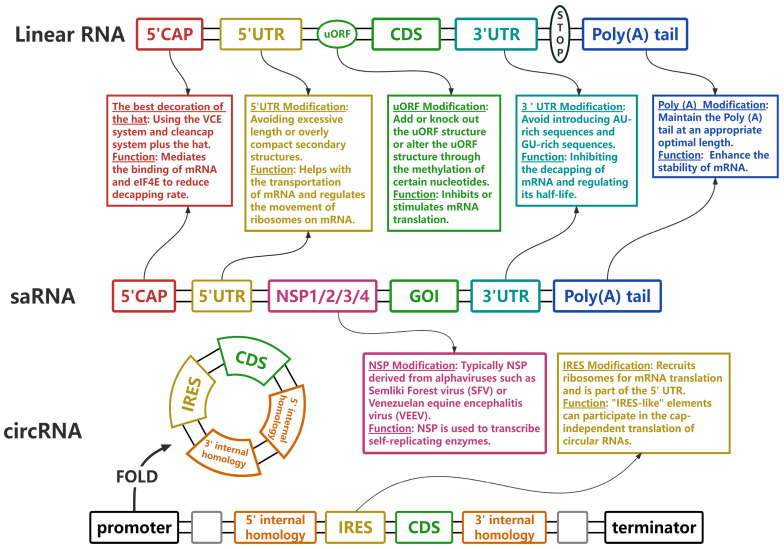
Diagram of the structures of three different types of mRNA vaccine. This section introduces the fundamental structures of three distinct types of mRNA and the various roles played by their components. By modifying and optimizing specific key elements, mRNA can enhance its efficiency and stability during the translation process. UTR: Untranslated Region, uORF: upstream open reading frame, saRNA: self-amplifying mRNA, circRNA: circular mRNA, CDS: coding DNA sequence, GOI: gene of interest, NSP: non-structural protein.

Generally, mRNA vaccines are classified as linear RNA, self-amplifying mRNA (saRNA), and circular mRNA (circRNA) (Figure 2). Traditional linear mRNA, due to its well-established research and comprehensive theoretical framework, has emerged as the most prevalent form, and plays a crucial role in gene expression. saRNA leverages viral replication elements to enable intracellular mRNA amplification, resulting in abundant protein expression, which can generate vaccines targeting any known protein pathogen, such as influenza, chlamydia, HIV-1, Ebola, and RSV. In contrast to the efficient expression of saRNA, circRNA features a covalently circular structure that is synthesized either through enzymatic methods or by the arrangement of self-splicing introns that connect the 3′ and 5′ ends. This unique structure confers greater biological stability and protects circRNA from degradation by exonucleases [7,8].

Although the three types of mRNA have distinct characteristics, their basic structures are fundamentally consistent, and during each mRNA structural elements interact with each other; each one also performs its own function based on various modifications to ensure the translation and integrity of mRNA.

#### 2.1.1. Cap Modification

The 5′cap of mRNA is recognized as endogenous RNA, protecting RNA molecules from degradation by 5′–3′ exonucleases and serving as an anchor for the recruitment of initiation factors that facilitate protein synthesis and translation.

**Figure 3:**
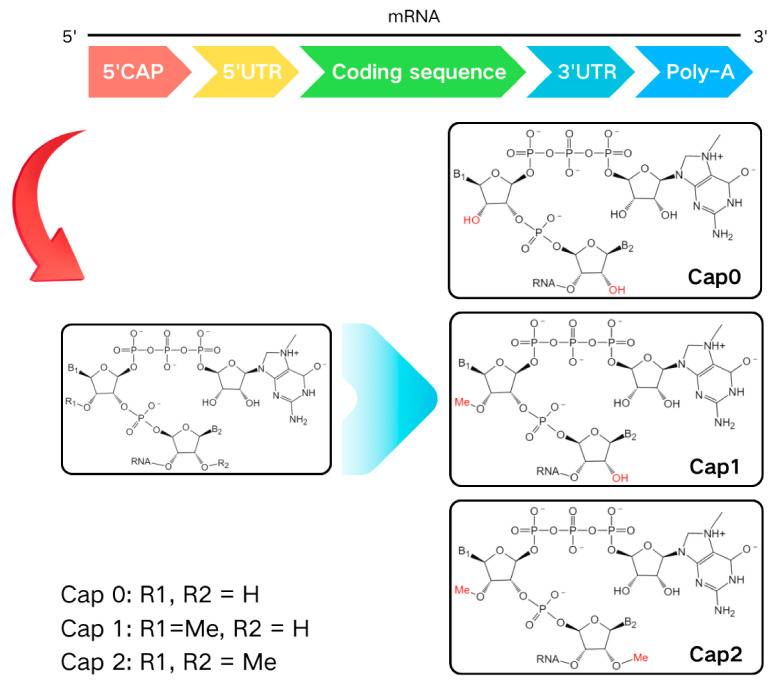
Chemical structure of 5′-cap of mRNA. In this specific RNA molecule, the methylation patterns presented by the initial few 5′ nucleotides are observed, and distinct cap variants such as CAP 0, CAP 1, CAP 2, and so on, can be clearly differentiated. The determination of these cap variants depends on the differences in the methylation patterns (as shown in the figure). B1, B2: nucleobases.

The N 7-methylguanosine (m^7^G) modification typically occurs at the 5′ cap of mRNA. This m^7^G cap enhances mRNA stability, facilitates transport, and is crucial for pre-mRNA processing and protein synthesis [9,10]. Depending on the methylation pattern of the initial few 5′-nucleotides in an m^7^G-capped RNA molecule, cap variants known as CAP 0, CAP 1, CAP 2, and so forth, can be identified [11] (Figure 3).

CAP 0 refers to mRNA that lacks additional methylation. The first nucleotide of all mRNAs is 2′-O-methylated (Nm) by the enzyme cap methyltransferase 1 (CMTR1), resulting in a CAP 1-modified mRNA terminal (m^7^G-ppp-Nm). Once mRNA enters the cytosol, cap methyltransferase 2 (CMTR2) performs 2′-O-methylation on a subset of CAP 1 mRNA at the second nucleotide, leading to the formation of the 5′ end of CAP 2-modified mRNA (m^7^G-ppp-NM-Nm). CAP 0 is commonly found in the mRNAs of many lower eukaryotes, whereas higher organisms generally exhibit more extensive methylation at their caps. CAP 2 can be present on all mRNAs, and, since it is generated after the mRNA, which has already acquired CAP 1, enters the cytosol, CAP 2 is enriched in long-lived mRNAs [12]. Research indicates that CAP 1 and CAP 2 in mammals assist cellular transcripts in evading the innate immune system and play significant roles in gene regulation.

The m^7^G of eukaryotic mRNA is formed in the nucleus, serving to protect the mRNA from degradation and promote the translation initiation [13]. A cap analog, based on the crystal structure complex of CAP-eIF4E, can directly label the solvent-exposed region of two-thirds of the m^7^G ribose. eIF4E exhibits a strong tolerance to this modification, thereby facilitating the efficient translation of capped RNA [14].

In addition to the m^7^G cap, there is the trimethyl-guanosine (TMG) cap, which is produced through the hypermethylation of the m^7^G cap [12]. This process involves the methylation of guanosine at the N7 position and is more prevalent in small nuclear RNA/small nucleolar RNA (sn/sno-RNA).

Two approaches for capping mRNA during in vitro translation (IVT) include the use of cap structure analogues for co-transcription and the application of a cap enzyme for post-transcriptional capping. Enzymatic capping requires multiple methylation steps and introduces two proteins, along with S-adenosylmethionine (SAM), making it a more complex procedure. On the other hand, co-transcriptional capping can be accomplished in a single step, allowing for direct purification following synthesis, which would hold greater promise in future novel mRNA vaccine design.

The first generation of cap analogues often yields low reaction rates and capping efficiencies for the cap 0 structure. To address this issue, clean cap technology, which represents a novel cap analogue, facilitates the incorporation of RNA polymerase into the CAP 1 structure. It can synthesize over 94% CAP 1 mRNA in a single-tube reaction and does not impose limitations on the concentration of triphosphate nucleotides (NTPs) during transcription, thereby enhancing the quality of full-length mRNA [15].

As our understanding of the 5′ cap deepens, we can expect a continuous emergence of diverse modification methods, which will provide a solid foundation for future research and development of mRNA-related drugs.

#### 2.1.2. Untranslated Region (UTR) Modification

As a non-coding region on mRNA, both the 5′UTR and 3′UTR play a critical role in maintaining RNA stability and regulating translation efficiency. The 5′UTR serves as an essential site for the ribosomal subunit to recognize the start codon, and its length can significantly influence ribosomal translation efficiency, particularly when maintained at intermediate lengths [16]. Study found that the N1-methylpseudouracil (m1Ψ) modification of mRNA can influence the translation efficiency of the 5′UTR.

However, the optimal 5′UTR of m1Ψ-modified mRNA (m1Ψ-5′UTR) leads to the development of Smart5UTR, which differs significantly from its unmodified counterpart; this is an all-in-one model algorithm that can be used to search for superior m1Ψ-5′UTR sequences. This innovative machine adopts a deep generative model to identify superior m1Ψ-5′UTR sequences computationally. By utilizing a customized loss function and network architecture, Smart5UTR can effectively design superior 5′UTRs, then greatly enhance mRNA vaccine development. Furthermore, an advanced 5′UTR vaccine targeting COVID-19 mRNA has been successfully developed utilizing the Smart5UTR design [17].

For the 3′UTR, Adenine–Uracil-rich elements (AREs) and Guanine–Uracil-rich elements (GREs) are closely linked to the stability of mRNA. ARE-binding proteins, such as tristetraprolin (TTP) and RNA-binding protein D (AUF1), can either stabilize or destabilize target mRNAs, depending on the location in the 3′UTR, thereby enhancing or inhibiting translation [18]. Elsewhere, the length of the 3′UTR has optimal length requirements, and mRNAs with longer 3′UTR have poor mean stability and short half-lives.

Additionally, RNA-binding proteins (RBPs) and microRNAs (miRNAs) regulate the stability and translational efficiency of mRNAs by binding to sequences in the 3′UTR [19,20]. Although research on RBPs in mRNA vaccines is extremely limited at present, considering their specific properties, it is reasonable to hypothesize that this particular characteristic of RBPs may play a certain auxiliary role in enhancing the efficacy of mRNA vaccines.

#### 2.1.3. Upstream Open Reading Frame (uORF) Modification

uORF consists of a start codon in the 5′UTR and an associated stop codon appearing before the stop codon of the main coding DNA sequence (CDS).

uORF primarily inhibits the translation of CDS through three mechanisms: leak scanning, ribosome stalling, and translation reinitiation. Leak scanning occurs when the ribosome bypasses the uORF and translates only the CDS, leading to the loss of the product component and adversely affecting translation. The ribosome stalling is attributed to the ribosome being retained upstream of the uORF, which diminishes the translation of the CDS. Translation reinitiation refers to the process of restarting a ribosome that is in the midst of translation, thereby suppressing further translation [21].

Furthermore, it has been shown that genes with different numbers of uORFs on the 5′ UTR have different sensitivities to loss of function. Genes with high sensitivities have more uORFs to regulate their translation efficiency [22].

#### 2.1.4. Poly(A) Tail (Polyadenylate Tail) Modification

Poly(A) tail can effectively enhance the stability and/or the translation efficiency of mRNA. This is attributed to poly-A-binding protein (PABP) facilitating the association of poly(A) tails with the 5′ cap via the translation initiation factors eIF4G and eIF4E. This interaction promotes the formation of a closed-loop structure, thereby enhancing translation efficiency.

The length of the poly(A) tail should be carefully considered, as its optimal length varies, depending on the specific host [23]. Early studies found that within 120 bp, the translation efficiency of the target antigen was elicited when the poly(A) tail increased, but the increased trend disappeared after the tail exceeded 120 bp [24]. Thus, we concluded that the optimal length of the poly(A) tail is best maintained between 100 and 120 bp [25]; this applies to dendritic cells derived from monocytes, among others. Subsequent research has demonstrated that in primary human T cells, poly(A) tails exceeding 300 nucleotides are more conducive to efficient translation. This phenomenon is associated with the trimming of poly(A) tails by PABP [26].

Methods for adding poly(A) tails can be categorized into two types: the first involves poly(A) polymerase-mediated polyadenylation of recombinant poly(A), while the second entails designing DNA templates for the direct transcription of poly(A) tails [26]. Although the latter method offers greater flexibility, it is also more cumbersome in laboratory operations. None of the above methods are suitable for large-scale production of mRNA, and the process of adding poly(A) tails requires further study.

#### 2.1.5. Nucleotide Modification

Current research indicates that modified nucleotides can effectively enhance the stability, translation efficiency, and immunogenicity of mRNA. Due to the operational flexibility and diversity of nucleotide modification, it has become a central focus among contemporary research. To date, over 50 modifications have been identified in various RNA molecules, with N6-methyladenosine (m6A) and m1Ψ being particularly prevalent in mRNA vaccines [27].

Cui Qi et al. employed gene knockout technology to reveal that m6A modification exerts a vital role in the self-renewal and tumorigenesis of glioblastoma stem cells (GSCs) [28]. Furthermore, substituting the uridine in in vitro transcribed (IVT) mRNA with a chemically modified derivative, such as pseudouridine (ψ), can diminish the intracellular adverse immune response to IVT mRNA. Remarkably, as a derivative of ψ, m1Ψ, used in the SARS-CoV-2 vaccines tozinameran (BioNTech-Pfizer) and elasomeran (Moderna), has been shown to reduce immune activation, improve mRNA translation, and produce higher levels than the Ψ Protein Yield. Another uracil analogue, 5-methoxyuracil (5 moU), has been shown to prolong IVT mRNA expression, thereby eliminating another major limitation of mRNA drugs. Simultaneously replacing uridine with 5 moU is sufficient to completely reduce immune activation and its associated functional impacts [29].

These findings show the significant practical implications of mRNA vaccine-based element modification and also promote the development of mRNA-based therapies. However, the biological functions and specific locations of these modifications in eukaryotic mRNA are poorly understood, and further studies are needed to improve their application.

### 2.2. mRNA Vaccine Delivery System

Wolff et al. confirmed the mRNA can be directly injected into mouse muscle for protein expression, in 1990 [5]; however, due to the intrinsic limitations of mRNA, including instability, susceptibility to degradation and strong immunogenicity, its development had been rather sluggish. Fortunately, the advent of delivery systems had effectively addressed many of the aforementioned issues and revolutionized the field of mRNA therapy.

With the help of the delivery device, mRNA shows multiple positive effects. Not only can it avoid nuclease attack, increase stability, and prolong half-life, but it also enables mRNA to enter the cytoplasm efficiently for protein synthesis. Furthermore, certain delivery means can lower the immunogenicity of mRNA, thereby mitigating inflammation and adverse reactions [30]. Up to now, the mainstream mRNA vaccine delivery systems can be mainly divided into two types: a natural or non-natural material-based mRNA delivery platform.

The natural material-based delivery system includes virus-like particles (VLPs), cell-penetrating peptides (CPPs), exosomes, etc. [31]. Exosomes have outstanding delivery capacity and a protective effect on mRNA molecules, with their low immunogenicity and strong targeting ability [32]. However, due to insufficient research on exosomes and potential biological risks, more extensive and long-term experiments are needed. Moreover, researchers are currently endeavoring to utilize microorganisms like bacteria and phages as novel delivery strategies to enhance the effectiveness of mRNA vaccines [33].

The non-natural material-based delivery system, such as LNPs, cationic nanoemulsions (CNEs) and polymer nanoparticles [34]. BNT162b2 [35], played an important role in combating the COVID-19 epidemic, and adopts the LNPs delivery platform. Nevertheless, despite the remarkable achievements, difficulties and challenges still persist. LNPs not only entail a high production cost, but also require strict low-temperature storage conditions to ensure their stability [36]. This may limit their promotion in worldwide, especially in some resource-scarce developing countries. So, the research and development of new delivery systems is the foundation for the transformation of mRNA vaccines.

### 2.3. mRNA Vaccine Immune Mechanism In Vivo

**Figure 4:**
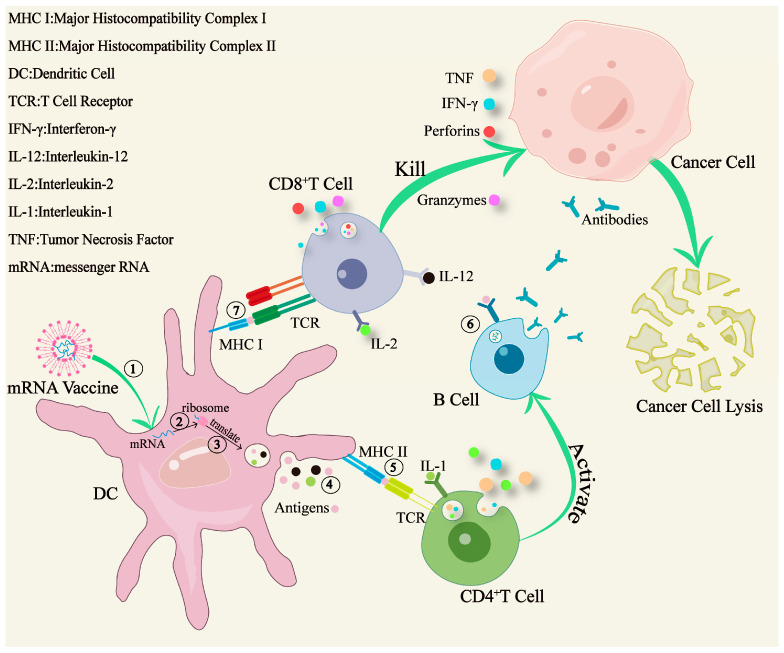
The immune mechanisms of mRNA tumor vaccines in vivo. The mRNA vaccine consisting of mRNA encapsulated by liposomes is absorbed by dendritic cells (DCs). Within DCs, mRNA undergoes the steps of transcription and translation to generate antigens, which are subsequently presented to T cells through MHC I or MHC II. Moreover, through the synergistic action of cytokines including interleukin-1 (IL-1), interleukin-2 (IL-2), and interleukin-12 (IL-12), the cellular immune pathway is activated. In particular, antigens secreted by APCs can also activate B lymphocytes. Under the action of activated CD4^+^ T cells, B lymphocytes will secrete corresponding neutralizing antibodies, further enhancing the immune effect on tumors. Immune activation progress: ① vaccine internalization, ② and ③ encoding protein, ④ protein delivery, ⑤, ⑥ and ⑦ activating immunity.

In comparison to traditional vaccines, mRNA vaccines typically require a sequential passage through several major pathways, following injection, to exert their function. These pathways include vaccine internalization, protein coding, protein transfer, immune activation, and cytotoxic cell activation (Figure 4).

As the first line of defense for the organism against pathogens, the innate immune response is activated at the earliest stage. The activation of the innate immune response in immune cells depends on the detection of pathogen-associated molecular patterns (PAMPs) by pattern recognition receptors (PRRs). Following vaccination, various PRRs identify mRNA and components of the delivery system as exogenous, resulting in the activation of the main Toll-like receptors (TLRs), which are primarily expressed on APCs [37]. TLRs, as a subset of PRS, play a crucial role in detecting PAMPs [38]. For example, the single-stranded RNA in mRNA vaccines can be recognized by TLR7/8, a specific type of Toll-like receptor. This recognition initiates a signaling cascade that leads to the activation of transcription factors such as nuclear factor kappa B (NF-κB) and interferon regulatory factors (IRFs) thereby inducing the production of pro-inflammatory cytokines like IL-6 and TNF-α, further enhancing the immune response [39].

mRNA-based vaccines can enter APCs via a delivery system to synthesize antigenic proteins and activate acquired immunity by presenting antigens. In addition to inducing T cell immunity, vaccines also promote the production of neutralizing antibodies, thereby further enhancing the immune response. For instance, BNT111 [40], an LNP-mRNA vaccine encoding melanoma-associated antigens, upon entering the lymph nodes, is ultimately engulfed by dendritic cells (DCs). Subsequently, DCs produce antigens and present them to T cells. CD8^+^T cells can recognize antigen peptides that are combined with MHC I. Following activation, they differentiate into cytotoxic T lymphocytes (CTLs) and directly kill tumor cells expressing the corresponding antigens. CD4^+^ T cells recognize and activate themselves through interactions with MHC II. Under the influence of tumor antigens encoded by BNT111, activated CD4^+^ T cells differentiate into Th1, Tfh and Treg cells, enhancing tumor eradication by secreting cytokines like IFN-γ and TNF. Furthermore, activated CD4^+^ T cells promote B cells to produce specific antibodies against tumors [41].

In tumor immunotherapy, researchers have discovered that the local delivery of mRNA-encoding pro-inflammatory cytokines enhances anti-tumor immunity. mRNA-2752 encodes two secreted cytokines (IL23 and IL36G), along with a membrane protein (OX40L). Phase I clinical trials are currently underway involving patients with advanced solid tumors, and early results indicate that mRNA therapy holds promise [42]. Additionally, a novel mRNA-based vaccine, ECI-006 [37,43], utilizes mRNA encoding various DC activation molecules and melanoma-specific tumor-associated antigens to augment cytotoxicity against tumors. The introduction of these new models presents innovative avenues for future research and development of tumor mRNA vaccines.

## 3. The Role of Tumor mRNA Vaccine and Its Clinical Research Status

With the emergence of novel delivery systems, neoantigens, and other innovative technologies, clinical studies on tumor mRNA vaccines have proliferated (Table 1). In the following sections, we will review the four most prevalent tumors and the corresponding role of tumor mRNA vaccines in anti-tumor therapy.

### 3.1. Hepatocellular Carcinoma

Based on data from the Global Cancer Observatory (GCO), hepatocellular carcinoma (HCC) accounts for 7.8% of cancer-related deaths, and is the third leading cause of cancer mortality [1].

To date, the mainstream treatments for liver cancer include surgical resection, transcatheter arterial chemoembolization (TACE), oral sorafenib, etc., but the results have been unsatisfactory [44]. Several clinical trials and studies have gradually substantiated the profound efficacy of mRNA-based liver cancer vaccines. The human hepatocyte nuclear factor 4 alpha (HNF4A) delivered via lipid nanoparticle messenger RNA (LNP-mRNA) can significantly mitigate the degree of liver fibrosis and restore hepatic cellular activity in mouse models [45]. Additionally, several Phase I clinical trials are currently underway to assess the therapeutic efficacy of mRNA-based vaccines specifically designed for the treatment of advanced HCC. For example, Pen X et al. attempt to use an anti-hepatitis B virus mRNA vaccine to improve the prognosis of hepatitis B virus-positive advanced-HCC patients [46]. This experiment is in the recruitment stage, without a definite clinical efficacy report yet. Meanwhile, Fan J et al. try to treat advanced-HCC patients by using the combination therapy of Stintilimab I injection and a neoantigen mRNA personalized cancer vaccine. The current experimental outcomes have demonstrated a tangible therapeutic potential for this modality [47].

All in all, the research and development of mRNA vaccines for HCC are advancing steadily. It is believed that an effective mRNA liver cancer vaccine will emerge, offering the hope a of cure to numerous patients suffering from this disease in the near future.

### 3.2. Lung Cancer

Lung cancer has the highest incidence and mortality in the world, and various strategies have been developed based on the activation of immune system [1]. Nowadays, leading mRNA vaccine firms CureVac and Moderna are developing mRNA-based lung cancer vaccines. Notably, CureVac’s CV9201 has progressed to Phase I/II clinical trials, due to its good tolerability and reasonable results [48]. As reported, CV9201 was well tolerated, with most adverse events including mild-to-moderate injection-site reactions and flu-like symptoms. Moreover, the mRNA vaccine encoding NY-ESO-1, MAGEC1, MAGEC2, MUC1, 5T4, and survivin (CV9202) was employed in combination with local radiotherapy for stage IV non-small-cell lung carcinoma patients. Potent antigenic responses were triggered in 84% of patients, and the immune intensity was at least twice that of the baseline. Notably, 42.6% of patients demonstrated stable disease as the best confirmed overall response. Following the combined therapy, local partial response was observed in one patient [49].

Thus, combining the lung cancer mRNA vaccine with local radiotherapy may promote the regression of lung cancer.

### 3.3. Melanoma

A total of 331,647 new cases and 58,645 deaths from melanoma were reported in 2022, the 17th largest number of new cases and 22nd largest number of deaths worldwide [1]. Immune checkpoint inhibitor (ICIs) therapy was the main immunotherapy treatment. Ipilimumab and tremelimumab are antibodies targeting cytotoxic T lymphocyte antigen 4 (CTLA-4). However, the combination of these two antibodies has been associated with an increased incidence of elevated aminotransferase levels and hepatotoxic events. Furthermore, ipilimumab adjuvant therapy was linked to a higher incidence of diarrhea, insomnia, and fatigue during the induction phase [50,51]. This adverse reaction between immune molecules in the body has limited its further application. Thus, the mRNA-based melanoma vaccine may be a new and valid choice.

At present, six clinical trials of mRNA-based melanoma vaccine are in progress and have promising outcomes [52] (Table 1). mRNA-4157/v940, in combination with pembrolizumab, constitutes a potential adjuvant therapy for high-risk melanoma patients who received combination therapy and who had a significantly reduced risk of disease recurrence compared to those who only received PD-1 inhibitor [53]. However, an mRNA nano-vaccine with a C1 lipid nanoparticle showed effective anti-tumor immunity in mice without significant harm to the body in 2021 [54]. Moreover, Ping, et al. identified five potential targeted tumor antigens (PTPRC, SIGLEC10, CARD11, LILRB1, and ADAMDEC1) [55], which are associated with overall survival (OS), disease-free survival (DFS), and APC function. Data analysis revealed that higher expression levels of these genes correlate with improved clinical prognosis for patients, providing a new direction for the subsequent development of mRNA melanoma vaccines.

Encouragingly, H Li et al. validated the fact that the circRNA-LNP platform demonstrated excellent efficacy in the treatment of B16 in in situ melanoma models, as well as in B16 lung metastasis models. Additionally, the platform exhibited synergistic antitumor effects when combined with adoptive T cell therapy in a model of advanced B16 melanoma in situ. These findings suggest that the circRNA-LNP vaccine has significant anti-tumor effects across various mouse tumor models [56]. In addition, previous studies have identified four types of antigens that can be utilized in the design of melanoma circular RNA vaccines: common shared antigens, including tyrosinase, Trp-2, chicken ovalbumin (OVA), and GP-100; common mutated antigens such as BRAF, KIT, and NRAS; cancer germline antigens like MAGE-A1, MAGE-A3, Bage, Gage, and NYESO-1; and newly expressed or acquired antigens, which include genomic mutations found in tumors that are not present in normal somatic cells [57]. Collectively, circRNA vaccines hold significant potential for tumor therapy and may serve as a promising alternative to linear mRNA vaccines.

### 3.4. Prostate Cancer

Prostate cancer is the most common malignant tumor in the male reproductive system. Based on the GCO, in 2022 the number of new cases of prostate cancer ranked fourth globally, with 396,792 global deaths [1].

Due to the adaptive changes of the tumor cells and mechanisms such as hormone resistance, patients are prone to develop drug resistance, resulting in a lack of appropriate treatment options for cure [58,59]. Hubert Kübler et al. evaluated the potential safety and immunogenicity of the CV9103 self-adjuvating mRNA vaccine in advanced prostate cancer patients in stages I/IIA. The vaccine can induce specific cellular and humoral immune responses, and a decrease in PSA and tumor shrinkage has been observed in some patients [60]. Moreover, KLHL17, CPT1B, IQGAP3, LIME1, YJEFN3, KIAA1529, MSH5, and CELSR3 are included as the potential antigen targets for mRNA vaccine development [61].

## 4. Conclusions and Prospects

The emergence of the mRNA vaccine has multiple forward-looking implications. Not only can it offer a swift response to emerging diseases, due to its rapid development and production capabilities, but it may also lead to the development of more effective cancer vaccines by stimulating the immune system with the help of TSA/TAA. Additionally, continuous research and development may drive innovation in the fields of biotechnology and pharmaceuticals, thereby opening up new pathways for disease prevention and treatment. However, opportunities always come with challenges. The transformation of mRNA drugs from basic research to clinical application still needs continuous research and progress. For instance, therapeutic tumor mRNA vaccines require higher doses and more injections, compared with preventive ones. Therefore, their safety assessment needs to be proven by longer-term clinical experiments. Future research should continue focusing on (but not be limited to) fully understanding and using the immune characteristics of mRNA vaccines, developing new delivery systems and new antigens to improve vaccine safety and stability.

## Figures and Tables

**Table 1:** Clinical trials of mRNA-based cancer vaccines.

NCT Numbers/Phase	Status	Disease	Combinations	Start Year
NCT02035956/I	Completed	Advanced Melanoma	NA	2013
NCT03289962/I	Active, not recruiting	Melanoma, NSCLC, Bladder Cancer,	With Atezolizumab	2017
NCT03815058/II	Active, not recruiting	Advanced Melanoma	With Pembrolizumab	2019
NCT03897881/II	Recruiting	Complete Resection of High-Risk Melanoma	With Pembrolizumab	2019
NCT04335890/I	Unknown status	Uveal melanoma	With standard therapy	2020
NCT01456104/I	Completed	Melanoma	NA	2011
NCT05738447/I	Recruiting	HCC	NA	2023
NCT01817738/I/II	Terminated	Prostate cancer	NA	2019
NCT01197625/I/II	Active, not recruiting	Prostate cancer	NA	2010
NCT03948763/I	Completed	CRC, NSCLC, pancreatic cancer	NA	2019
NCT00923312/I/IIa	Completed	NSCLC	NA	2009
NCT01915524/Ib	Terminated	NSCLC	NA	2013

Trials with recruitment status “Recruiting” and “Completed” were found on ClinicalTrial.gov. NSCLC: non-small-cell lung cancer, CRC: colorectal cancer, NA: not available.

## Data Availability

No new data were created or analyzed in this study. Data sharing is not applicable to this article.
